# TTM 2.0

**DOI:** 10.1186/1471-227X-15-S1-A4

**Published:** 2015-06-24

**Authors:** Christian Storm

**Affiliations:** 1 Department for Internal Medicine, Nephrology and Intensive Care, Cardiac Arrest Center of Excellence, Charité-Universitätsmedizin Berlin, Germany

## 

The professional treatment of the reperfusion syndrome following cardiac arrest and successful resuscitation has improved over the past decade. In 2002 and 2003 mild hypothermia treatment was first recommended by international guidelines following remarkable milestone trials. With growing experience and an increasing number of hospitals implementing hypothermia as standard treatment, technical devices also improved to enhance patient safety. The term target temperature management (TTM) has been introduced by several societies indicating that cooling down is not enough [1]. We are supposed to manage temperature therapy as part of a package of interventions after cardiac arrest [2,3]. As intracranial pressure can correlate with brain and body temperature, modern TTM is about precision although the optimal target temperature is still unknown. This has been shown by the current TTM Trial by Nielsen and colleagues comparing 33°C and 36°C as different target levels without a significant difference in outcome variables [4]. As these results have been criticized in several points, the conclusion can only be that obviously different patient characteristics need a more individual TTM following cardiac arrest. As stated, TTM 2.0 indicates we have reached the next step that includes professional TTM in a large bundle of therapy steps such as early coronary intervention, close monitoring of supportive care and precise neurological prognostication. The way to go is still long as TTM is still underused worldwide [5,6].

If TTM would be seen as a medication or drug, it should be adjusted or dosed depending on the severity of the disease or hypoxic brain damage. Especially, a moderate reperfusion injury will respond and benefit from a medication like TTM compared with a severe and long-lasting hypoxia leading to death despite any therapy. Therefore, a key in the near future will be the early grading of the hypoxic brain damage by a combination of clinical data, biomarkers, advanced neurological monitoring and radiological findings. This might lead to a more personalized and adjusted post-cardiac arrest treatment.

## Financial disclosure

CS has received honorarium and/or travel costs from Medivance, Zoll, C. R. BARD, Philips, EMCOOL, COVIDIEN and Nonin; and financial or material support for research from Medivance, Zoll, C. R. BARD, EMCOOL, COVIDIEN, Nonin and Deutsch Stiftung für Herzforschung.

## Declaration

This abstract and supplement was proposed, developed and commissioned by BMC Emergency Medicine and was funded by an educational grant from C. R. BARD, NJ, USA. The published abstract was independently prepared. C. R. BARD had no input into the content.
